# Mental health service engagement with family and carers: what practices are fundamental?

**DOI:** 10.1186/s12913-021-07104-w

**Published:** 2021-10-09

**Authors:** Darryl Maybery, Irene Casey Jaffe, Rose Cuff, Zoe Duncan, Anne Grant, Melissa Kennelly, Torleif Ruud, Bjorg Eva Skogoy, Bente Weimand, Andrea Reupert

**Affiliations:** 1 Monash University, Clayton, Australia; 2 Harvard University, Cambridge, USA; 3 Satellite Foundation, Melbourne, Australia; 4 Queen’s University Belfast, Belfast, UK; 5 Akershus University Hospital, Nordbyhagen, Norway; 6 Nordland Research Institute, Bodø, Norway; 7 University of South-Eastern Norway, Notodden, Norway

**Keywords:** Family, Carers, Health services, Engagement, Practices

## Abstract

**Background:**

Substantial and important benefits flow to all stakeholders, including the service user, when mental health services meaningfully engage with carers and family members. Government policies around the world clearly supports inclusiveness however health service engagement with family and carers remains sporadic, possibly because how best to engage is unclear. A synthesis of currently used surveys, relevant research and audit tools indicates seven core ways that families and carers might be engaged by health services. This study sought to confirm, from the perspective of family and carers, the importance of these seven health service engagement practices.

**Methods:**

In a mixed method online survey, 134 family members and carers were asked what they received and what they wanted from mental health services. Participants also quantified the importance of each of the seven core practices on a 0–100 point likert scale.

**Results:**

Almost 250 verbatim responses were deductively matched against the seven themes, with additional unaligned responses inductively categorised. The findings triangulate with multiple diverse literatures to confirm seven fundamental engagement practices that carers and family want from health services. Conceptually, the seven practices are represented by two broad overarching practice themes of (i) meeting the needs of the family member and (ii) addressing the needs of the service user.

**Conclusion:**

Policy, clinical practice, training and future research might encompass the seven core practices along with consideration of the intertwined relationship of family, carers and the service user suggested by the two broader concepts.

**Supplementary Information:**

The online version contains supplementary material available at 10.1186/s12913-021-07104-w.

## Background

In many western countries’, the deinstitutionalisation of services has shifted the focus of support from hospitals to the community, where carers and family members play a vital role in supporting those experiencing mental health issues [1]. Family members may provide emotional, social and instrumental support to an individual and work collaboratively with services developing and delivering treatments [2, 3]. In the U.S.A., 43.5 million adults were carers of another in 2014 with 60% being female and with many carers experiencing burden and reduced ability to work due to their caring for a family member [4]. In 2015, 4 % of Australians were carers of mental health service users with 39% caring for their partner, their parent (31%) or their child (18%) with primary carers providing an average of 40 h per week of care [5]. Twelve percent of those carers were aged 15 to 24 years of age.

Involving families in health care has clear benefits for the person receiving treatment [2, 6], can reduce the burden often experienced by families and carers [7–9] and address families’ social and emotional needs [10]. Governments worldwide have recognised the importance of family and carers in policy and practice guidelines. Policy documents include the National Institute for Clinical Excellence guidelines for the treatment of schizophrenia in the United Kingdom [11], the National Guidelines on Relatives in the Health-and Care Services in Norway [12], the PORT Guidelines in the United States [13], the Royal Australian and New Zealand College of Psychiatrists clinical practice guidelines for the management of schizophrenia and related disorders [14] and in the World Health Organisation Authority Mental Health Action Plan 2013–2020 [15]. This paper is concerned with translating these guidelines and policy documents into practice by conceptualising the fundamental ways that family and carers might engage with and/or be engaged by health services.

However, there can also be major negative impacts from providing care. Hill & Broady summarise the literature in suggesting that carers can feel isolated, emotionally burdened, have high levels of distress and low personal wellbeing and sometimes feeling resentful due to their caring role [10]. It has been suggested that health services involving families in the care of their ill relative is at best variable, but often poorly implemented and inadequate [7]. In a large carer survey in the US for example only 32% of carers were asked by “… a doctor, nurse, or social worker … about what was needed to care for their recipient” (p.12) and only 16% were asked about their own needs [4]. In the UK, a 2015 survey of family members found that less than 35% of participants reported being taken seriously or listened to by the mental health services and even fewer were provided with the information and advice they needed [16]. Family members frequently report a lack of involvement in planning and often feel excluded from the treatment process [17–19]. Others have suggested that family and carer burden may be partly induced by being excluded from the collaborative treatment of their loved one and the inadequate support for their own needs [20–23]. A crucial question concerns how should health services collaborate with family members and carers [24]?.

Equally, most research into family engagement and involvement has primarily focused on clinicians’ opinions and experiences about working with families [23, 25–27] and not the views of family members themselves [27]. Research from the family perspective acknowledges that many family and carers are already partnering the service user in their care (e.g. Weimand et al. [4, 22]) and that this should be acknowledged by services and involve families in decisions about care and discharge [28].

‘Family’ is used here to include those with a significant personal relationship with the mental health service user. This might include biological relatives, intimate partners, ex-partners, people in co-habitation, children, friends, those with kinship responsibilities, and others who play a significant role in the service user’s life. References to family may focus on the family of origin (an individual’s parents and other family members) and/or family of procreation (an individual’s children and other family members) [29]. A carer, who does not need to be a blood relative, is defined as “… someone who is actively supporting, assisting or providing unpaid care to a consumer … having … a significant role in the life of … someone … who has received, is receiving, or is seeking, treatment and support from … health services.” [30] (p. 1–2). However, the term carer has been criticised as it can convey dependency and negates the reciprocal give and take relationship that exists between some carers and the consumer [31]. Moreover, “some family members may identify themselves as a ‘carer’ in a consumer’s^1^ life, others will identify more so with the characteristic of their relationship, for example, parent, child, partner, sibling” [30]. Additionally, while some family members care for their relative, they do not necessarily identify as ‘carers’ [32]. Nonetheless, ‘carer’ is a term that governments use and many subscribe to [33]. In this paper we use both family and carer terms, aiming to elicit information from both carers and family about their relationships with health services.

Table 1 summarises relevant survey instruments, research papers and audit tools that detail how carers and family members might engage with or be engaged by mental health services. To determine the core areas of engagement with carers and families we drew where possible on literature reviews (e.g. Foster and colleagues) but also upon grey and research literatures that highlight the research practice interface. This included survey instruments and practice audits that highlight engagement practice with family and carers by service providers. The first column in Table 1 highlights seven practices that were found across many of the survey instruments, practice audit tools and research literature.

**Table 1:** Summary of family and carer focused practices from survey instruments, concepts of practice from the literature and practice audit tools

Family and carer practices	Survey instruments			Research			Practice audit tools	
	Carer experience survey [34]	FFP workforce questionnaire [35]	CUES-C [36]	Practice standards [37]	FF practice review [38]	Conceptual-ising FFP [39]	File audit [40]	Think family audit [41]
**1. Identify and acknowledge family and carers**	Access and Engagement			Acknowledge Parenthood status (including carer and children)			Acknowledge Parenthood status (including carer and children)	Acknowledge Parenthood status (including carer and children)
**2. Engage and communicate with family and carers**	Access and Engagement.	Engagement issues		Engaging with carer or children	Coordinated system of care between family & services	Genuine communication	Engaging with carer or children	Engaging with carer or children
**3. Involve family member/carer in planning/ collaboration**	Involvement in Treatment Identification of Carers. Planning for Ongoing Care		Involvement in planning of treatment and care	Recognition/input of carer and children into planning (e.g. service user recovery, risk plan	Family care planning & goal setting	Collaborative care and treatment planning	Invite input of carer and children into treatment planning	Invite input of carer and children into treatment planning
**4. Assessment of family member/ carer needs**		Assessing the impact on the child Connectedness		Strength/Risk assessment to children/ Dependent	Assessment of family members		Strength/Risk assessment to children/ dependent	Strength/Risk assessment to children/ dependent
**5. Provide or offer ongoing support to family and carers**		Support to carers and children Parenting and mental illness	Support for carers	Family support	Instrumental, emotional & social support	Practical support to the family	Family support	Family support
**6. Provide psycho-education to family and carers**		Family and parenting support	Information about mental illness and its effects	Psychoeducation	Psycho-education to family members	Awareness raising and education	Psycho- education	Psycho- Education
**7. Provide or recommend referrals of family and carers to another service**		Provide referrals		Use of other services for carer or children	Liaison between family & services		Use of other services regarding needs of carer or children	Use of other services regarding needs of carer or children

### Survey instruments

Survey instruments are one way that policy and practices have been conceptualised to capture the experiences of carers and/or families. They are often used by services for practice evaluation purposes, from various stakeholder perspectives. For example, the Australian Carer Experience Survey [34], designed for carers, is based on a review of the academic and grey literatures and the National Standards for Mental Health Services [42]. Several core practices are identified from this survey (see Table 1). Two other instruments are the UK CUES-C, designed to measure carers’ experience across domains that carers see as important in the care of the service user [36] and the Family Focused Workforce Questionnaire [35]. The latter measure has been widely used [27, 43–47] and is designed to measure, from the perspective of clinicians, the extent to which they engaged with family members, provided support to family members, addressed issues related to parenting with a mental illness, provided family members with referrals to other services and assessed the impact of the parent’s illness on the child (again see Table 1 for relevant engagement practices).

### Research

From the broad research literature, the term “family focused practice” (FFP) has commonly been used by service users, family members and carers as well as researchers, policy makers and practitioners to operationalise the ways in which services and families and carers might collaborate [48]. Reupert et al. [39], employed a community participatory approach with service users, family members (parents, children, siblings, partners), and those that identify as carers, clinicians, and managers, to conceptualise FFP. FFP was operationalised as genuinely communicating with family members, engaging family members in collaborative care and treatment planning, providing practical support to the family, awareness raising of the issues involved with mental illness and delivering education about mental illness to the community. Once again, these core engagement concepts are summarised in Table 1. Drawing on 40 research papers conducted in adult, child and adolescent mental health settings, Foster et al. [38], conducted an integrative literature review to synthesize the underlying concepts of FFP. Six core practices were identified; (i) a coordinated system of care between the family and services, (ii) family care planning and goal setting, (iii) providing instrumental, emotional and social support, (iv) delivering psycho-education to family members, (v) liaising between family and services and (vi) assessing the needs of family members. Expanding on available research literature, Goodyear et al. [37], also reviewed policy documents, interviewed senior practitioners, and employed a Delphi approach with practitioners to operationalise family inclusive practice standards for adult mental health services, with a particular focus on families where a parent has a mental illness. Standards included acknowledging parenthood status, engaging with family members including children, ensuring that family members and children have input to treatment planning, collaborating with other services, providing a strength/risk assessment of children and providing support and psychoeducation to family members.

### Practice audit tools

Audit tools have also operationalised family and carer engagement, with the aim to systematically examine routine clinical practices in health settings. For example, the Think Family audit tool, designed for use in adult mental health and children’s services in Northern Ireland [41], involves identifying the presence of children and the parenting role of service users, engaging with carers and children, recognising the input of carers and children into planning (e.g. risk plan), providing support and psychoeducation to family members, collaborating with other services when responding to the needs of family members, and assessing the strengths and risks for children. Similar practices are audited in the Australian Standards of Practice and Audit Tool to audit practices related to families where parents have mental health issues [40]. Notably, the Irish and Australian audit tools were independently conceptualised but measure very similar practices or concepts.

The survey instruments, concepts of practice from the research literature and practice audit tools can be synthesized into a framework with seven core practices to engage family and carers (Table 1). These include:

1. Identify and acknowledge family and carers;
2. Engage and communicate with family and carers;
3. Involve family and carers in planning/collaboration in consumer’s treatment;
4. Assess vulnerable family member or carer’s needs;
5. Provide or offer ongoing support to family and carers;
6. Provide psycho-education to family and carers, and
7. Provide or recommend referrals for family and carers.

It is critical to determine if these seven core practices align with family members and carers’ preferences and accurately reflect the types of practices, they consider to be important. Hence, this study sought to confirm the applicability and importance of the seven practices from the perspective of family and carers.

The two aims of this mixed method study were (i) to elicit the engagement practices that family members and carers want from mental health services and (ii), to have carers and family quantify (rate) the importance of the seven core practices. From these findings we sought to determine the fundamental practices that family and carers want when they engage with mental health services.

## Method

### Participants and recruitment

One hundred and thirty-four participants with a mean age 34.01 years (sd 17.93 ranging from 18 to 81 years) participated in this study. This included 11 males (8.2%) and 121 females (90.3%) from overwhelmingly English-speaking households (98.5%) with 26.9% secondary educated, 41.8% with TAFE or some University qualification and 29.9% with a University qualification. Sixty three (47%) were family but did not consider themselves to be in a caring role and of the 71 (53%) who did 3 (2.2%) had been caring for 0–12 months, 28 (13.4%) 1–5 years, 22 (16.4%) 5–10 years and 28 (20.9%) 10 years and longer. The average age of the service user was 45.54 years ranging from 6 to 81 years with 56 (41.8%) males. The service user relationship to the participants was spouse/partner 17 (12.7%), parent 86 (64.2%) and son or daughter 25 (18.7%). Almost half of the participants indicated that their service user had multiple diagnoses. The main mental health problems of the service users were anxiety, depression, substance use and schizophrenia.

Participants were invited to participate through social media, referral or word of mouth and directed to a dedicated Facebook page that provided general information about the study and a link to an information sheet and the questionnaire.

### Design

The study utilised a mixed method design employing a modified version of the van Bon-Martens [49] concept mapping procedure to integrate practical and scientific knowledge by selecting participants with relevant experiences, designing the questions on concepts, collecting data on identification and rating of concepts, and analysing the results from practical experiences and scientific knowledge. The van Bon-Martens procedure was chosen because it was a systematic and suitable method for integrating the experience from carers and family with scientific knowledge from the literature.

At the start of the online questionnaire participants completed demographic items about themselves and the service user (see Results section for details). Participants self-identified as family or carers by responding (Yes/No) initially to the question *Are you a family member of the patient/consumer?* Regarding being a carer they then responded (Yes/No) *Do you consider yourself a carer for the patient/consumer*? To maintain anonymity participants did not provide identifying information and consent was assumed if they completed the online questionnaire. The next part of the questionnaire consisted of the two qualitative open-ended questions. It is important to note that at this point participants answered these open questions before receiving any information on the seven identified core practices. The third and final part consisted of the quantitative questions to rate the importance of each of the seven core practices, and the participant were not able to change their answers on the two previous open ended questions after they had been exposed to the seven core practices.

### Ethics approval and consent to participate

All methods were carried out in accordance with relevant guidelines and regulations. All experimental protocols were approved by Monash University via ethics review number 18074. Informed consent was obtained from all participants and none were under 18 years of age.

#### Data collection, instrument and data analyses

In the first instance, participants were invited to respond to the following open question:

*What have been the most important things the patient’s health service or health services workers have done for you as a family member or carer? Please list at least 3.*

After answering they were then asked:

*What other things would you have liked the patient’s health service or health service workers to have done for you as a family member or carer?*

It should be noted that each participant was invited to offer more than one response to these open questions. This resulted in many more responses than there were individuals in the study (i.e. 134 participants provided 249 responses to question 1). For this stage, data were analysed deductively, by aligning responses to the open-ended text to the seven themes. Responses that could not be aligned with the seven themes were analysed independently by two of the authors, to identify potential additional practices.

The next stage of the survey focused on the seven family focused practices shown in Table 1 where participants were invited to rate the relative importance of each. For example participants were asked to *please indicate from 0 = not important to 100 = extremely important …* (1) *How important is it for health services to identify and acknowledge carers and family of the patient (health service user)?*

## Results

### Qualitative responses- deductive analysis

There was a total of 249 responses to the question, ‘*what have you as a carer/family member received’* and 223 responses from the question, ‘*what they would have liked to have received’*. These qualitative responses were examined independently by two authors to deductively classify responses according to the seven family focused practices (shown in Table 1). This sought to confirm any or all of the seven practices and identify any additional practices. Responses not aligned to the seven summary practices, were qualitatively analysed by the two authors using Braun and Clarke’s [50] six step process. This process involves (i) data familiarisation by reading and rereading transcripts, (ii) generating initial codes, (iii) searching for themes amongst code patterns (iv) reviewing potential themes in relation to the coded data and entire data set, (v) defining and labelling themes and finally, (vi) the production of a report [50]. Braun and Clarke’s approach is a systematic well validated approach to qualitative analysis.

Of the 249 responses to the initial question, 157 were classified according to the seven themes. For the second question, 134 of the 223 responses were classified in relation to the seven themes. Table 2 shows the seven themes from the literature in column 1 and the remaining columns illustrate the number and percentage of responses classified from the two questions, along with verbatim examples.

**Table 2:** Classification of responses to open questions ‘What did family/carers get and what did they want from mental health services’ according to seven core themes

Theme	*What did you get?*		*What did you want?*	
	*#* *%*	Examples of verbatim comments	*#* *%*	Examples of verbatim comments
1. Identify and acknowledge family and carers	15 6%^a^	*Acknowledge and welcome me to the ward.* *Made me feel comfortable during visitation.* *Listened.*	5 2%	*Be more compassionate and understanding.* *Listened to the family more.* *To have me as a carer involved.*
2. Engage and communicate with family and carers.	25 10%	*Keeping me informed. As a carer for my mum at a young age for a long time I wasn’t properly informed on what was going on or what she was going through, when this changed as I got older it helped a lot in me being able to provide care.* *Ask me about his needs and routine. Talk to me about his progress.* *Proactive communication.*	32 14%	*Better communications is essential.* *Listen to me as his carer not ignore me.* *Been more inclusive of family (*sic*) members & their information & opinions & had a professional discussion inclusive of the patient prior to discharge.*
3. Involvement in planning/ collaboration in service user’s treatment	9 4%	*With my dad (patient) s consent- involved the family in planning his care.* *Have case conferences.* *Included us in the patient’s health plans.*	4 2%	*Involved the family in treatment.* *Respect and, information on discharge to manage person at home.* *Very important for family to have input - family see the patient over the whole day, 7 days per week.*
4. Assessment of vulnerable family member or carer’s needs	7 3%	*Understanding the ways it affected me and asking important questions about my safety under her care.* *Ask about my mental health, due to impact of caring role.* *Looking out for the safety of me and my siblings.*	9 4%	*To look after me more. No one seemed to care about how my mother’s illnesses were impacting on me especially when I was a child.* *Checked in with me regarding my own health and wellbeing.* *Suggested services that might be helpful for my own health and wellbeing.* *Care for how this was affecting me.*
5. Provide or offer ongoing support to family and carers	66 27%	*Got my younger brother and I to come in for an appointment with his psychologist when we were younger.* *Given support to myself and my husband.* *Respite care service if required*	42 19%	*Reassured me as a child that I wouldn’t be taken away just because she had problems, because overall, she wasn’t a bad mother.* *More ongoing counselling services* *help with supporting me to deal with my own emotions and heart ache when my child has episodes.*
6. Provide psycho-education to family and carers	22 9%	*Helped the family with how to respond to problems my mum* *Communication re the diagnosis and treatment in simple english avoiding medical jargon.* *Support services and they were very supportive of me as a parent and gave me lots of valuable advice to help me manage my son.*	30 13%	*More education and communication about the condition and how to care for her at home,* *I would like to have met someone qualified so they could explain to me what is happening and what are my risks in the future for having a mental illness.* *When talking in acronyms explain what it means and not in a rushed manner.*
7. Provide or recommend referrals for family and carers	13 5%	*Linked me in with support services so I had someone to talk to about the effects it was having on my mental health.* *Ensured that I had support/respite and explained the next steps.* *Helped me to help my son, find services.*	12 5%	*Providing links for other services available especially in finance.* *More respite and help in accessing what help and services are on offer.* *Offer referral for counselling.*

^a^Percentages are calculated based upon **all** responses to each question (ie against 249 and 223 responses respectively)

Notably, there were some discrepancies between the two authors whose views most commonly diverged between classifying items on themes 1 and 2 and then, 5 and 7. Divergences stemmed from different interpretations of the meaning of the comment and/or in relation to the meaning of the theme. For example, *I was in young carers, I had access to resources such as psychologists* and *Kept in touch with me,* and *Allowed space between family members, referrals* and *Respite care service if required* was assigned by one author to **5. Provide or offer ongoing support to family and carers** and the other author assigning the comment to **7. Provide or recommend referrals for family and carers.**^2^ It was not clear whether the health service was providing the additional support service or referring the family/carer to the service. The two authors acknowledge the interpretation overlap but came to an agreement based on verbatim comment classifications that were later acknowledged by and agreed to by the broad author group.

### Qualitative responses- inductive analysis

Approximately one third of the responses could not be categorised according to the seven themes. Many of these comments 43 (17%; *what have you received*) and 59 (29%; *what they would have liked*) were focused upon the family and carers’ views on the qualities of the treatment of the service user. This focus on quality of treatment included multiple subthemes related to the treatment or service that the consumer received. These included the importance of consistency of treatment (*case manager … ongoing … not short term*); that there be more services for consumers (*provide mental health support*), be more active with the consumer (*not leave them sitting in emergency for 42 h before being seen by a Dr*.) and to provide Better treatment/services (*stop prescribing and providing advice in isolation*) and provide other services (e.g. *arrested him, make sure that he is showered every day and clean*). Another group of items was that carers and family members had received nothing from the mental health service. Thirty-nine (16%) comments included such things as, “*I have not received any support whatsoever from the patient’s health service or health service workers”* and *“nothing nothing nothing*”. There were also a small number of responses that could not be categorised elsewhere 10 (4%) and 7 (3%) respectively. The former included some positive feedback such as, “*They are doing a great job”.*

### Quantitative analysis of responses

The second aim of this study was for participants to quantify the importance of the seven core practices on a 0 (Not important) to 100 (Extremely important) scale. Table 3 (column 2) shows the means (SD) for each with average scores ranging from 88.70 for **provide and/or recommend referral of family members and carers to other services** to a high of 92.18 for **to engage and communicate with carers and family members of the patient (health service user)**. Participants considered all seven to be highly important practices.

**Table 3:** Statistics (Mean, Standard Deviation on a scale of 0 = Not important to 100 = Extremely important), Pearson correlations and principal component loadings and variance explained for the seven themes quantitative data (*n* = 134)

Quantitative items *How important is it for health services to …*	Mean (SD)	Pearson Correlations						Component Loadings	
		1	2	3	4	5	6		
(1) ..identify and acknowledge carers and family membersof the patient (health service user)?	91.48 (11.71)							**.89**	.04
(2) ..engage and communicate with carers and family membersof the patient (health service user)?	92.18 (10.43)	.71^**^						**.93**	.11
(3) ..collaborate with family members and carers in the care planningof the patient (health service user)?	89.47 (14.16)	.53^**^	.48^**^					**.53**	−.32
(4) ..assess the strengths and vulnerabilities of family members and carers regarding their own health including mental health and wellbeing?	90.42 (14.58)	.32^**^	.28^**^	.51^**^				−.10	**−.94**
(5) ..provide or arrange for support to carers and family membersof the patient (health service user)?	88.88 (15.37)	.40^**^	.46^**^	.48^**^	.70^**^			.10	**−.85**
(6) ..educate family members and carers about the patient’s(health service user’s) health condition?	91.22 (13.51)	.40^**^	.42^**^	.36^**^	.25^**^	.36^**^		**.62**	−.08
(7) ..provide and/or recommend referral of family members andcarers to other services (including service for the carer or familymental health)?	88.70 (15.16)	.41^**^	.33^**^	.39^**^	.62^**^	.73^**^	.40^**^	.05	**−.84**
**Total**	**90.34** **(13.68)**	**Variance Explained**						**53.51**	**16.50**

** Significant at *p* > .01

In recognition of the above-mentioned disagreement on deductive classification of responses to some of the seven themes (i.e. 1 and 2 and 5 and 7) we then examined the quantitative responses further for relationships between how participants had scored the seven practices. We acknowledge this to be ‘opportunistic’ but given the mixed method data collected, it was considered conceptually important to examine the quantitative data further. Table 3 displays the correlations between the seven variables, showing stronger correlations between variables 1 and 2 and 5 and 7, along with relatively strong correlations between item 4 with 5 and 7.

Principal component analysis [51] was then undertaken with the seven items to determine if there was a higher order structure to the items. Initially a Kaiser-Meyer-Olkin test was undertaken to determine if the data were suited for component analysis. The .78 KMO result suggested the data were adequate and a principal component analysis with oblique rotation was then undertaken on the seven items. The analysis converged in 8 iterations with a clear two component structure that is illustrated in Table 3 by the loadings in the last two columns with 70.01% of variance being explained by the analysis.

Consistent with the correlation between items 1, 2, 3 and 6 loaded together as did items 4, 5 and 7 in the component analysis. This quantitative analysis is also consistent with the divergent view of items being classified between themes 1 and 2 and between themes 5 and 7.

## Discussion

Together the qualitative and quantitative findings from this study support the seven family and carer engagement practices identified from previous survey instruments [34–36], research reviews and studies [37–39] and file audit tools [40, 41]. Together this suggests that these seven engagement practices are fundamental ways that family and carers want to be engaged by mental health services.

The qualitative analysis of unprompted participants’ comments confirmed the seven family focused practice areas as both what carers and family had received and what more they would have liked, from mental health services. The provision of ongoing support to family and carers was most commonly nominated by participants (27% *received* and 19% *wanted*), however, all seven engagement practices were highlighted by study participants as what they received and wanted from mental health services. These qualitative data were then triangulated in the second part of the study, with participants scoring each of the seven practices as very to extremely important engagement practices. The findings confirm recommendations from policy in the USA from the Patient Outcomes Research Team (PORT) guidelines that recommend support to families [13]. Likewise, these findings confirmed psychiatry policy in Australia and New Zealand which recommend that “… effective support for families is crucial …” for the service users mental health and to reduce family burden due to the illness [14] and the Norwegian Health Directorate in their specific development of National Guidelines on Relatives in the Health-and Care Services [12]. When updating family and carer policy in future, governments should closely consider the application of these seven practices to service provision.

The findings also highlight clear gaps in family and carer survey instruments. For example, psychoeducation is missing on the Carers Experience Survey [34], involving carers in planning is not represented on the FFP Workforce questionnaire [35] and a lack of acknowledgement and engagement with family and carers is absent from the CUES survey [36]. However, the recently developed file audits [41, 52] and practice standards [37] do reflect the seven core themes as do the six key areas of literature summarised by Foster and colleagues in the family focused practice review [38]. The latter are not surprising as they guided our understanding of workforce practices in Table 1. Nevertheless, the qualitative and quantitative information collected from participants in this study support these practices as being fundamentally important. However, it needs to be emphasized that carer and family involvement in the support and treatment of the service user has to be on their terms, not because they feel obliged or see the need to address a gap that services should fill. Likewise, their contributions should not replicate existing services but supplement, extend and support services. Their place as equal but different to what services can provide to service users, needs to be acknowledged and then further involvement negotiated.

An unexpected finding was the conceptual overlap between the seven engagement practices. This was initially evident with the divergence of researcher opinion when classifying verbatim content and then quantified statistically by stronger correlations between items 1, 2, 3 and 6 (see Table 3 for items) and between items 4, 5 and 7, suggesting that those practices are conceptually similar. The subsequent exploratory component analysis also indicated two broader or higher order [51] factors. Consequently, we tentatively propose that carers and family engagement with services has two functions at a higher hierarchical level, focused on achieving ‘benefits for the service user’ and ‘benefits to carers/family members’ (see proposed structure and 7 practice definitions (modified from Kennelly [53] in Additional file 1). Similarly, in a qualitative survey study with 216 relatives, Weimand et al. [20] found two broad themes, one titled “Striving for involvement for the sake of the mentally ill person,” which focused on relatives concerns that the service user was involved in services, and the other “Wanting inclusion for the sake of oneself”, which highlighted the need for their own engagement in services. Likewise, these findings suggest that at a higher conceptual level, health service engagement with carers and family might serve two functions, namely, providing ‘benefits for the service user’ and providing ‘benefits to carers/family members’. The findings also suggest that the two higher concepts are closely connected. When services engage family and carers services are able to provide better treatment/follow-up of the service use. In turn, this may reduce the burden of care of the service user and so, reduce the family member’s need for support or care regarding their own needs.

A concerning response from a minority (16%) of participants was that they had received ‘nothing’ from the mental health services. While concerning, it is not overly surprising given that less than a third of carers (e.g. in the U.S.A.) are asked about the service users or their own needs [4] and in the UK similar numbers reported not being taken seriously, listened to or provided with information by the mental health services [16]. Given that policy and guidelines, in some cases for many years [13], have advocated for the recognition of carer and family involvement in services, this finding suggests that mental health services have much work to do. One solution might be that family and carer roles may need to be clearly defined in legislation, with follow up professional development, supervision and support. This paper gives clear direction to governments and health services that they enshrine these seven fundamental approaches in their policies, and that they expect these practices from health service provision with carers and families. The participants in this study clearly confirmed that the seven practices are how they would like to be engaged by health services. Further, the tentative finding of two higher order concepts, suggests that policy and practice should consider both the requirements of the service user as well as the requirements of the carer and family members.

### Strengths and limitations

There are several limitations to this research including the modest number of self-selecting participants who were mostly female (90.3%) and from English-speaking backgrounds (98.5%). Broader sampling might highlight different patterns of responses to the themes (i.e. some carer groups may appreciate alternate specific types of responses from services) or even suggest additional responses. These appear fruitful areas for future research. While a strength of the study was the mixed method design, the quantitative instrument was developed by the authors without attention to psychometric properties. Other questions such as what practices were most helpful may have been better stem questions (e.g. alternatives to importance) about the seven practices.

## Conclusions and implications

The findings broadly support the seven core practices for services and conceptually suggest two higher order foci for attention. The future development of policy, audit tools, carer survey instruments and service practice measures or standards should consider these seven core areas of practice. Future research could examine these questions and investigate further the lower and higher order structures suggested by the findings. Other fruitful areas of future research might examine if these seven engagement practices have application to different types of carers/relatives (parents, children etc) and in relation to other service user illness groups (e.g. substance problems, aged care, cancer). Finally, and arguably most importantly, service users need to be asked how they want family members and carers involved, to ascertain their views on these seven practices.

## Supplementary Information

**Additional file 1.**

## Data Availability

Requests for data should be made to Darryl Maybery (darryl.maybery@monash.edu), Monash University, Australia (Corresponding author).

## Footnotes

1 Throughout the document we have used the term ‘service user’ rather than consumer to refer to the individual receiving health service support for their mental health condition. At times the term consumer is quoted from references.

2 During the journal process of manuscript review all quotes assigned to the 7 practices and additional inductively assigned data can be made available to the journal editor and reviewers.

## References

[1] Lobban F, Postlethwaite A, Glentworth D, Pinfold V, Wainwright L, Dunn G, Clancy A, Haddock G (2013). A systematic review of randomised controlled trials of interventions reporting outcomes for relatives of people with psychosis. Clin Psychol Rev.

[2] Pharoah F, Mari J, Rathbone J, Wong W (2010). Family intervention for schizophrenia. Cochrane Database Syst Rev.

[3] Lucksted A, McFarlane W, Downing D, Dixon L, Adams C (2012). Recent developments in family psychoeducation as an evidence-based practice. J Marital Fam Ther.

[4] The National Alliance for Caregiving and the AARP Public Policy Institute. Report: caregiving in the U.S; 2015. p. 11. https://www.aarp.org/content/dam/aarp/ppi/2015/caregiving-in-the-united-states-2015-report-revised.pdf. Accessed 15 July 2020.

[5] Productivity Commission (2020). Mental Health, Report no. 95, Canberra.

[6] Pitschel-Walz G, Leucht S, Bäuml J, Kissling W, Engel RR (2001). The effect of family interventions on relapse and rehospitalization in schizophrenia-a meta-analysis. Schizophr Bull.

[7] Hsiao CY, Lu HL, Tsai Y (2019). Factors associated with primary family caregivers’ perceptions on quality of family centered care in mental health practice. J Nurs Scholarsh.

[8] Maybery DJ, Goodyear MJ, Reupert AE, Harkness MK (2012). Goal setting within family care planning: families with complex needs. Med J Aust.

[9] Okpokoro U, Adams CE, Sampson S (2014). Family intervention (brief) for schizophrenia. Cochrane Database Syst Rev.

[10] Hill T, Broady T (2019). Understanding the social and emotional needs of carers: final report (social policy research center report 2/19).

[11] National Institute for Health and Care Excellence (NICE) (2014). Psychosis and schizophrenia in adults: treatment and management. NICE clinical guideline 178.

[12] The Norwegian Directorate of Health. [Helsedirektoratet] (2017). National guidelines on relatives in the health and care services [Pårørendeveileder. Veileder om pårørende i helse- og omsorgstjenesten].

[13] Dixon LB, Dickerson F, Bellack AS, Bennett M, Dickinson D, Goldberg RW, Lehman A, Tenhula WN, Calmes C, Pasillas RM (2010). The 2009 schizophrenia PORT psychosocial treatment recommendations and summary statements. Schizophr Bull.

[14] Galletly C, Castle D, Dark F, Humberstone V, Jablensky A, Killackey E, Kulkarni J, McGorry P, Nielssen O, Tran N (2016). Royal Australian and New Zealand College of Psychiatrists clinical practice guidelines for the management of schizophrenia and related disorders. Aust N Z J Psychiatry.

[15] Mental Health Action Plan 2013–2020. World Health Organisation. 2013. Downloaded on 5/03/21 from https://www.who.int/mental_health/publications/action_plan/en/.

[16] Care Quality Commission (2015). Right here, right now: mental health crisis care review | care quality commission.

[17] Doody O, Butler MP, Lyons R, Newman D (2017). Families’ experiences of involvement in care planning in mental health services: an integrative literature review. J Psychiatr Ment Health Nurs.

[18] Keogh B, Skärsäter I, Doyle L, Ellilä H, Jormfeldt H, Lahti M (2017). Working with families affected by mental distress: stakeholders’ perceptions of mental health nurses educational needs. Issues Ment Health Nurs.

[19] Skärsäter I, Keogh B, Doyle L, Ellilä H, Jormfeldt H, Lahti M (2018). Advanced the knowledge, skills and attitudes of mental health nurses working with families and caregivers: a critical review of the literature. Nurse Educ Pract.

[20] Weimand BM, Hedelin B, Hall-Lord ML, Sällström C (2011). Left alone with straining but inescapable responsibilities: relatives’ experiences with mental health services. Issues Ment Health Nurs.

[21] Weimand BM, Sällström C, Hall-Lord ML, Hedelin B (2013). Nurses’ dilemmas concerning support of relatives in mental health care. Nurs Ethics.

[22] Weimand BM, Hall-Lord ML, Sällström C, Hedelin B (2013). Life-sharing experiences of relatives of persons with severe mental illness – a phenomenographic study. Scand J Caring Sci.

[23] State of Victoria. Royal Commission into Victoria’s Mental Health System, Final Report, Summary and recommendations, Parl Paper No. 202, Session 2018–21 (document 1 of 6). 2021.

[24] Landeweer E, Molewijk B, Hem MH, Pedersen R (2017). Worlds apart? A scoping review addressing different stakeholder perspectives on barriers to family involvement in the care for persons with severe mental illness. BMC Health Serv Res.

[25] Grant A, Reupert A (2016). The impact of organizational factors and government policy on psychiatric nurses’ family focused practice with parents who have mental illness, their dependent children and families in Ireland. J Fam Nurs.

[26] Eassom E, Giacco D, Dirik A, Priebe S (2014). Implementing family involvement in the treatment of patients with psychosis: a systematic review of facilitating and hindering factors. BMJ Open.

[27] Grant A, Reupert A, Maybery D, Goodyear M (2018). Predictors and enablers of mental health nurses’ family-focused practice. Int J Ment Health Nurs.

[28] Giacco D, Dirik A, Kaselionyte J, Priebe S (2017). How to make carer involvement in mental health inpatient units happen: a focus group study with patients, carers and clinicians. BMC Psychiatry.

[29] Reupert A, Maybery D, Nicholson J, Gopfert M, Seeman MV, editors. Parental psychiatric disorder: distressed parents and their families. 3rd ed. Cambridge University Press; 2015. 10.1017/CBO9781107707559.

[30] Victorian Government. Working together with families and carers: chief Psychiatrist’s guidelines. Victorian Government: Department of Health and Human Services; 2018.

[31] Molyneaux V, Butchard S, Simpson J, Murray C (2010). Reconsidering the term ‘carer’: a critique of the universal adoption of the term ‘carer’. Ageing Soc.

[32] Henderson J (2001). ‘He’s not my carer—he’s my husband’: personal and policy constructions of care in mental health. J Soc Work Pract.

[33] Seal K, Murray C, Seddon L (2015). The experience of being an informal “carer” for a person with cancer: a meta-synthesis of qualitative studies. Palliat Support Care.

[34] Commonwealth of Australia. Mental health carer experience survey. Australian Government: Australian Health Ministers Advisory Council Australian Mental Health Outcomes and Classification Network; 2016.

[35] Maybery D, Goodyear M, Reupert A (2012). The family focused mental health practice questionnaire. Arch Psychiatr Nurs.

[36] Lelliot P, Beevor A, Hogman G, Hyslop J, Lathlean J, Ward M (2003). Carers’ and users’ expectations of services – carer version (CUES-C): a new instrument to support the assessment of carers of people with a severe mental illness. J Ment Health.

[37] Goodyear M, Hill T-L, Allchin B, McCormick F, Hine R, Cuff R, et al. Standards of practice for the adult mental health workforce: meeting the needs of families where a parent has a mental illness. Int J Ment Health Nurs. 2015;2(2):169. 10.1111/inm.12120–80. 25619407

[38] Foster K, Maybery D, Reupert A, Gladstone B, Grant A, Ruud T, Falkov A, Kowalenko N (2015). Family focused practice in mental health care: an integrative review. Child Youth Serv.

[39] Reupert A, Ward B, McCormick F, Ward C, Waller S, Kidd S (2018). Developing a model of family focused practice with consumers, families, practitioners and managers: a community based participatory research approach. BMC Health Serv Res.

[40] Goodyear M, McDonald M, von Doussa H, Cuff R, Dunlop B (2018). Meeting the intergenerational needs of families where a parent has a mental illness. J Parent Fam Ment Health.

[41] McCartan C, Bunting L, Davidson G, Devaney J, Donaghy M, Duffy J, Grant A (2020). Think family NI audit in adult mental health and children’s services.

[42] Commonwealth of Australia (2010). National standards for mental health services.

[43] Reupert AE, Maybery DJ, Morgan B (2015). The family-focused practice of primary care clinicians: a case of missed opportunities. J Ment Health.

[44] Grant A, Goodyear M, Maybery D, Reupert A (2015). Differences between Irish and Australian psychiatric nurses’ family focused practice in adult mental health services. Arch Psychiatr Nurs.

[45] Skogøy BE, Ogden T, Weimand B, Ruud T, Sørgaard K, Maybery D (2019). Predictors of family focused practice: organisation, profession, or the role as child responsible personnel?. BMC Health Serv Res.

[46] Leonard R, Linden M, Grant A (2020). Predictors of family focused practice among health visitors: a mixed methods study. J Adv Nurs.

[47] Ueno R, Maybery D, Reupert A, Osada H (2020). Translation and validation of the family-focused mental health practice questionnaire-Japanese version. Int J Ment Health Promot.

[48] Skogøy BE. From policy to practice: Implementation of changes in law to support and protect children of ill parents. PhD thesis. 2019. https://hdl.handle.net/10037/17328.

[49] Bon-Martens M, Goor I, Holsappel JC, Kuunders T, Bruggen M, Te Brake H, Oers H (2014). Concept mapping as a promising method to bring practice into science. Public Health.

[50] Braun V, Clarke V (2006). Using thematic analysis in psychology. Qual Res Psychol.

[51] Gorsuch RL (1983). Factor analysis.

[52] Victorian FaPMI coordinators - Practice Standards Working Group. Development of the FaPMI file audit tool. Identifying families at intake – a summary of approaches in Victorian mental health services, Best Practice/Next Practice Forum. 2018. https://www.bouverie.org.au/support-for-services/fapmi/fapmi_events/best_practice_next_practice_forum. Accessed 15 July 2020.

[53] Kennelly M. Development of a file audit tool to measure the involvement of family and carers in public mental health service. Monash University: Masters Thesis, Monash University; 2019.

