# Supplementary material for: Mental health service engagement with family and carers: what practices are fundamental?
Source: BMC Health Serv Res. 2021 Oct 9;21:1073. doi: 10.1186/s12913-021-07104-w (PMC8502279; doi:10.1186/s12913-021-07104-w)
Supplement: Supplementary file 1 — Additional file 1. [file 12913_2021_7104_MOESM1_ESM.docx]

Additional file 1: Higher and lower order day to day fundamental health service engagement practices and definitions.

| **Fundamental health service responses** | | **Definition*** |
| --- | --- | --- |
| **Broader (higher order) practices** | **Day to day (lower order) practices** |  |
| 1. **Benefit most focused on the service user**   ***Definition:*** The health service relationship with the carer or family is usually focused upon recovery benefits of the service user. | 1. Identify and acknowledge family and carers | Reflects an early and accurate enquiry, recording and an ongoing review of the individuals who are within the family system of the service user. This includes the type of role and relationship to the service user. This standard appreciates the legislated rights of all stakeholders within the family to be included and also to decline inclusion in a service uer’s care. |
|  | 2. Engage and communicate with family and carers | Requires genuine and open communication with family members and carers. Entails some flexibility in approaches be made to develop and foster these relationships. There is an ongoing information exchange occurring. |
|  | 3. Involvement in planning/ collaboration | Multiple perspectives are sought out, listened to, explored and utilised in order to support the service user’s care and recovery. Family and carers are considered to be active partners in developing and implementing aspects of care. |
|  | 6. Provide psycho-education to family and carers | Information and education that is relevant and useful, and is based upon what family and carers want to know. Psychoeducation in general aims to improve family members’ mental health literacy and can range from informal discussions through to manualised family interventions. |
| 1. **Benefit most focused on the carer or family member**   ***Definition:*** The health service relationship with the carer or family provides them with assessment and support that benefits their wellbeing. | 4. Assessment of vulnerable family member or carer’s needs | Identifying and screening around safety needs, strengths and limitations that may be present for individual family members or within the family as a whole. The level of these needs can vary considerably. Risks to any dependent children or young people are a particular concern in this definition. |
|  | 5. Provide or offer ongoing support to family and carers | Practical and emotional assistance that is given or recommended for the family in response to the family and carer’s individual circumstances and needs. |
|  | 7. Provide or recommend referrals for family and carers: | Encouragement and assistance that is given or suggested to family and carers, to set up a connection or appointment for appropriate and more specialised help. This includes health professional advocating with services to achieve better outcomes for families. |
| * Adapted from Kennelly^53^ | | |
